# Parapatric subspecies of *Macaca assamensis* show a marginal overlap in their predicted potential distribution: Some elaborations for modern conservation management

**DOI:** 10.1002/ece3.4405

**Published:** 2018-09-04

**Authors:** Madan K. Suwal, Falk Huettmann, Ganga Ram Regmi, Ole R. Vetaas

**Affiliations:** ^1^ Department of Geography University of Bergen Bergen Norway; ^2^ EWHALE Lab University of Alaska Fairbanks Fairbanks Alaska; ^3^ Global Primate Network Nepal GPO BOX 26288 Kathmandu Nepal

**Keywords:** Asia, Assamese macaque (*Macaca assamensis*), Himalaya, MaxEnt, Random Forest, species distribution models

## Abstract

Phylogenetic niche conservatism implies that sister taxa will have similar niches, although the niches of disjunct subspecies may evolve differently. This study uses *Macaca assamensis,* subspecies *assamensis* and *pelops,* to investigate the similarities of realized climatic niches of two disjunct subspecies (separated by the Brahmaputra River) along with a similarity analysis of their respective regions’ climate. Modeled distributions were used to quantify their potential distribution under current and future climate scenarios. The climatic similarity between regions of each subspecies was tested with principal component analysis (PCA), and the realized climatic niche overlap between two subspecies was tested with a multivariate analysis of variance (MANOVA) on a subset of the least correlated variables out of 24 publicly available topo‐bioclimatic variables. Tukey's honest significance difference (HSD) was used to test the range differences (on all 24 variables) between subspecies. The potential distribution of both taxa in the current climate and projected future climate was model‐predicted using MaxEnt and Random Forest. We found significantly different climatic ranges for 21 predictors (HSD;* p *<* *0.05) for the two subspecies, significantly different climatic conditions for their regions (using PCA;* p *<* *0.001), and significantly different realized climatic niches for the two subspecies (MANOVA;* p *<* *0.001). The distribution models generated a larger potential area than the currently known distributions. Although literature show that the Brahmaputra River is an effective dispersal barrier, we found some of the neighboring geographic range for both subspecies appears to be potentially suitable for the other taxon. The projected future potential areas indicate that some parts of the currently occupied geography, mostly southern parts, may become climatically unsuitable, whereas other new geographical areas may become suitable. Most of these new potential areas will be toward the north where higher and fragmented mountains, which has conservation implications.

## 1 INTRODUCTION

Phylogenetically closely related sister species and subspecies are expected to show similarities in their niches (Losos, 2008; Peterson, 2011; Peterson, Soberón, & Sánchez‐Cordero, 1999). However, there is some empirical evidence that contradicts this expectation (Chen, Hill, Ohlemüller, Roy, & Thomas, 2011; Peterson & Holt, 2003). Subspecies that live in different geographical locations (allopatric distribution), or in different zones along mountain slopes, may have different niches (Nakazawa et al., 2010; Vetaas, 2000). Niche is an n‐dimensional environmental space (fundamental niche) which is constricted (realized niche) by species interactions, dispersal limitations, and land use (Hutchinson, 1957; Sax, Early, & Bellemare, 2013; Zhao, Ren, Garber, Li, & Li, 2018). “Climatic niche” is the climatic space occupied by a species in a realized geographic distribution (Peterson et al., 2011).

Species distribution can be characterized by climatic variables including precipitation and temperature, their interaction, and topography (Bell, Bradford, & Lauenroth, 2014; Margules, Nicholls, & Austin, 1987); these variables are part of the principal dimensions of a species’ fundamental niche (Hutchinson, 1957). The principal dimensions of fundamental niches tend to overlap between closely related species and subspecies, as suggested by phylogenetic niche conservatism (Losos, 2008; Peterson et al., 1999). However, this concept is complex and cannot be studied and expressed well with parsimony (Drew & Perera, 2011) because the realized niche of a species has many more determining factors such as predator–prey relationships, food availability, disturbance, and other behavioral and ecological processes, in addition to climatic variables (Cushman, Littell, & McGarigal, 2010; Hutchinson, 1957). Ecological niche models (ENMs) without such range‐constraining factors do not really represent the “true” realized niche of a species. Species distribution models (SDMs) based on such ENMs with only topo‐climatic variables tend to produce a potential distribution, rather than the realized geographic distribution of species (Jiménez‐Valverde, Lobo, & Hortal, 2008; Sax et al., 2013).

The climate forecast in the “business‐as‐usual” scenario (defined as future development trends following those of the past without any change in policy (Metz, 2001)), also known as Representative Concentration Pathways (RCP) 8.5, projects the average surface temperature to be 2.6°C to 4.8°C warmer by the end of this century compared with the 1986 to 2005 period (Collins et al., 2013). The change in average surface temperature and precipitation regime may generate a novel climate in the future (Collins et al., 2013; Pendergrass & Hartmann, 2014; Williams, Jackson, & Kutzbach, 2007). A globally coherent “fingerprint” of current climate change impacts on species has been recorded by different meta‐analyses (Chen et al., 2011; Parmesan & Yohe, 2003), and similar impacts on species are projected under future climate conditions (Bedia, Herrera, & Gutiérrez, 2013; Peterson et al., 2002; Zhang et al., 2015).

Most studies on niche similarity are carried out within species and between species, and between hybridizing parents and their decedent species (Nakazawa et al., 2010; Peterson et al., 1999; Suwal & Vetaas, 2017; Vetaas, 2002). The intraspecies (e.g., subspecies) fundamental niches are expected to overlap to some extent, because fundamental niches are conserved over time (Losos, 2008). However, intraspecies realized niches may differ because of geographic isolation (such as allopatric or parapatric distribution), dispersal limitation, and competitive exclusion (Garcia‐Ramos, Sanchez‐Garduno, & Maini, 2000).

**Figure 1:**
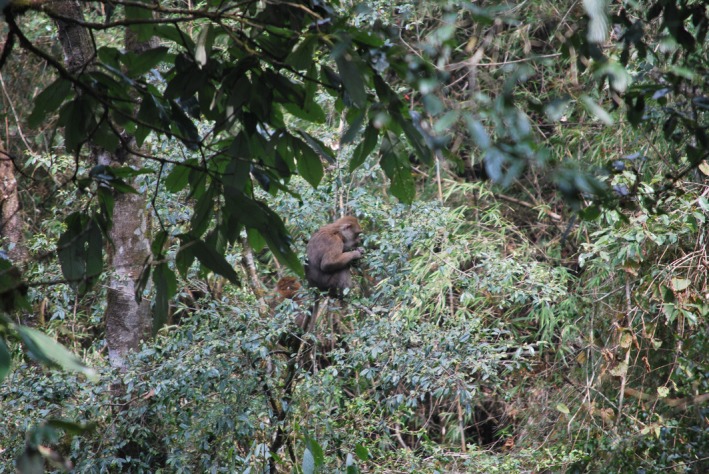
Western Assamese macaque (*Macaca assamensis pelops*) in the moist broad‐leaved forest of eastern Nepal, at elevation approx. 2, 700. Photograph by coauthor GRR

The primary aim of this study was to predict the potential climate niche for *Macaca assamensis* M'Clelland 1840 (Assamese macaque, Figure 1) based on species distribution modeling*. Macaca assamensis* diverged from *M. radiata* when *M. radiata* expanded its distribution from the Indian peninsula towards the Himalayas (Fooden, 1988). *Macaca assamensis* has since been divided into two subspecies; *M. assamensis* ssp. *pelops* (western population) and *M. assamensis* ssp. *assamensis* (eastern population; further taxonomic details can be found in the “Taxa” section; Fooden, 1988; Roos et al., 2014). We study the within‐species climatic niches and distribution overlap of these two subspecies of *Macaca assamensis* under the current climate and under a future projected climate using topo‐climatic variables. The eastern population became isolated from the western population (source) because of glacial retreat in a warm period during the late Pleistocene to Holocene, which transformed the glacier into a major river, creating the current barrier at the eastern end of the Himalayan mountain chain (Fooden, 1988; Khanal et al., 2018). This gives rise to the question: Do the subspecies have a high degree of similarity in their realized climatic niches, as explained by phylogenetic niche conservatism (Losos, 2008; Peterson et al., 1999), or do they have differently realized climatic niches because of climatic context from disjunct distributions since the last maximum glaciation (ca. 18,000 years ago)? To answer this question*,* here we set out to investigate (a) whether climatic conditions are similar between the respective regions of the two subspecies of *M. assamensis*, (b) whether quantified realized climatic niches are similar between the taxa, and (c) where the potential distributional areas under current and future climate scenarios are located?

## 2 METHODS

### 2.1 Study area

**Figure 2:**
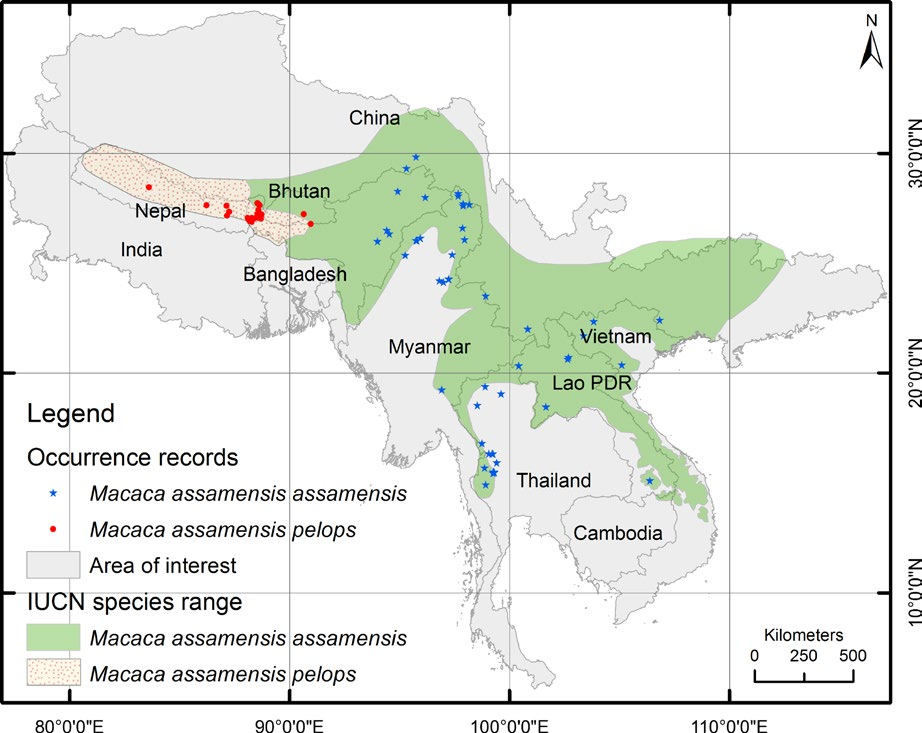
Map of the study area and recorded locations of eastern and western populations of *Macaca assamensis*. The IUCN range map was extracted from the IUCN Red List portal (http://www.iucnredlist.org), accessed on 27 November 2016

The study area ranges between 77°E to 117.3°E and 5.6°N to 36.5°N and covers most of the Hindu‐Kush Himalayan region including Nepal, Bhutan, Bangladesh, Myanmar, Laos, Thailand, Cambodia, Vietnam, as well as northern parts of India and southern parts of China (Figure 2). We considered a study area larger than the currently known distribution of *Macaca assamensis* (Fooden, 1980, 1988; Roos et al., 2014; Timmins & Duckworth, 2013; Wada, 2005), so that models may reveal any peripheral potential areas that are not yet known. Given such a large study area, there is high topographical diversity including floodplains, valleys, gentle to steep mountain slopes, and small streams to very large rivers.

Most of the study area is dominated by monsoon climate, where a high proportion of the precipitation occurs during summer with a minor cycle of precipitation during the winter (Yihui & Chan, 2005). The eastern region has more evenly distributed precipitation throughout the year compared to the western region (http://sdwebx.worldbank.org/climateportal). The study area offers tropical, subtropical, and temperate climatic regions as well as alpine.

Rapid urbanization and extension of agriculture in the last few decades have led to considerable transformation of forest in this region (Giri, Often, Pradhan, Kratzschmar, & Shrestha, 1998; Zhao et al., 2006), making agriculture the dominant land‐use type in the region (Stibig et al., 2007). This transformation has fragmented the habitats of the macaque species (Boonratana, Chalise, Das, Htun, & Timmins, 2008).

### 2.2 Taxa

Assamese macaque (*Macaca assamensis* Integrated Taxonomic Information System Taxonomic Serial Number (TSN) 573018) is a member of the *sinica* group. It is categorized as “Near Threatened” in the Red List compiled by the International Union for Conservation of Nature (IUCN). The species inhabits the mountain regions of the central and eastern Himalaya, and adjoining south and southeast Asian mountain chains (Boonratana et al., 2008; Fooden, 1980, 1982). The eastern population is said to range from Arunachal Pradesh in India to Thailand, Laos, Vietnam, and the Yunnan and Guangxi provinces in China; the western population is described as being distributed from West Bengal in India to central Nepal (Groves, 2001). Two subspecies of *M. assamensis* are distinguished as the southeast Asian *Macaca assamensis* ssp. *assamensis* (TSN 945194) and the sub‐Himalayan *Macaca assamensis* ssp. *pelops* (TSN 945195). The two parapatric subspecies are separated by a described zoogeographical barrier (defined here as a physical obstacle that prevents migration of *M. assamensis*); the Brahmaputra river in northeastern India (Fooden, 1982; Roos et al., 2014). The distribution of the two subspecies is fairly well‐known, but quantitative mapping and the characteristics of their niche and distribution are lacking (Regmi et al., 2018).

### 2.3 Occurrence data and pseudo‐absence data

We used open‐access species occurrence data from Regmi et al. (2018; collection from 1998 to 2013) and Fooden (1982; museum records from 1849 to 1980; *n* = 186 for *M. assamensis* ssp. *pelops* and *n* = 184 for *M. assamensis* ssp. *assamensis*) to generate the models. These are “occurrence only” data, and we therefore generated background data randomly throughout the study area at a minimum of 5 km linear distance (to avoid possible clustering of multiple points; 5 km is an arbitrary value equivalent to approximately five times the pixel size, which is meaningful in landscape scale) between any two points in ArcGIS 10.3 (ESRI; total background data points = 39,884) to produce the niche models and the species distribution models.

### 2.4 Environmental variables

On a broad scale, species distribution is usually correlated with the two principal climate factors: precipitation and temperature (Bell et al., 2014; Thomas, 2010), which also applies to mammals (Li et al., 2013). In addition, different ecological processes such as predator–prey dynamics and food availability also govern mammalian species distributions (Li et al., 2013; McPherson & Jetz, 2007; Trainor, Schmitz, Ivan, & Shenk, 2014). Theoretically, all ecological processes are required for an informed study of the realized niche and realized distribution, but potential niche and potential distributions can be estimated based on just climatic variables (Bobrowski, Gerlitz, & Schickhoff, 2017; Bobrowski & Schickhoff, 2017; Drew & Perera, 2011).

The predictive performance of species distribution depends also on the magnitude of climate change as well as partly on the choice of input data and their resolution (Bobrowski & Schickhoff, 2017; Trivedi, Berry, Morecroft, & Dawson, 2008). Hence, as a test we used two different sources of bioclimatic variables: Climatologies at High resolution for the Earth's Land Surface Areas (CHELSA; Average of 1979–2013; Karger et al., 2016, 2017) and WorldClim (Version 1.4, average of 1960 to 1990; Hijmans, Cameron, Parra, Jones, & Jarvis, 2005). This allows for a comparative study similar to the one done in Bobrowski and Schickhoff (2017). Both climate datasets are derived from the same source of data with a few but fundamental differences in their preparation. The CHELSA dataset is more recent and meant to be an improvement derived from statistical downscaling with gains in mountain regions, whereas the earlier WorldClim dataset is based on weighted spatial interpolation and widely established (Bobrowski & Schickhoff, 2017). This study is among the few, which have started to use the CHELSA data in a comparative fashion. The CHELSA data have only recently been released, whereas WorldClim has been in use for more than a decade unchanged. The CHELSA data are not fully tested yet by the global user community, but claim to correct a weakness of WorldClim data in orographic precipitation values (Karger et al., 2016). Orographic precipitation correction is particularly important for studies modeling species distributions in mountainous areas such as the Himalaya (Bobrowski et al., 2017; Singh & Kumar, 1997). The variables with CHELSA data will be called “CHELSA‐predictors,” and the variables with WorldClim data will be called “WorldClim‐predictors” hereafter.

Here, we used 24 predictors, which include 21 bioclimatic variables (bio01 to bio19, annual biotemperature (ABT; Holdridge, 1947; Li, Wen, Guo, & Du, 2015), the Ellenberg climatic quotient (EQ; Ellenberg, 1988; Mellert et al., 2016); ABT and EQ have a consistent time period and resolution with other bioclim variables) as well as three topographic variables (Supporting Information Table S1). The topographic variables are elevation (SRTM 90 m digital elevation model [Jarvis, Reuter, Nelson, & Guevara, 2008]), derived slope, and aspect in ArcGIS 10.3 (ESRI). Although land cover is an important variable in the distribution of species, its unavailability for future periods meant we did not include it in our model preparation. Climatic variables, however, should compensate for its absence. The high‐resolution topographic data were not aggregated to match the coarse climate data because we did not use the raster file but instead used a point‐based method where raster values were extracted to points and analyzed (Kandel et al., 2015; Regmi et al., 2018). All the data used in this study are open access, and the variables we prepared (ABT, EQ, slope, aspect) as well as occurrence data have been made open access via a university repository http://hdl.handle.net/1956/16960.

In a traditional approach, one of the problems when working with multiple variables is multicollinearity (Alin, 2010), which is reduced by omitting highly correlated variables (Elith, Kearney, & Phillips, 2010; Fox & Weisberg, 2010). Here, we followed this approach and to assess which variables were highly correlated, variable clusters were plotted using the *varclus* function (Harrell, 2013) in *R* (R Core Team, 2017) for both CHELSA‐ and WorldClim‐predictors (Supporting Information Figure S1). We also calculated variance inflation factors (VIF) for all variables using the *R* package *usdm* (Naimi, Hamm, Groen, Skidmore, & Toxopeus, 2014). We selected one variable with the smallest VIF value among the farthest cluster members from each cluster. When there was a single variable in a clade, the variable was also selected. This resulted in 17 CHELSA‐predictors and 15 WorldClim‐predictors. Next, the variance inflation factor (VIF) function (*vifstep)* in the *usdm R* package was used to select the final list of least correlated variables, using a threshold of VIF < 5.0 (Guisan, Thuiller, & Zimmermann, 2017). This gave seven common and two specific variables for both CHELSA‐ and WorldClim‐predictors (Supporting Information Table S1). These subsets of variables were used to analyze realized climate niche differences between taxa and generate species distribution models.

### 2.5 Future climate scenario selection for potential distribution

The global warming trend in the past century, particularly the last few decades, has been at a higher rate compared to previous centuries (IPCC, 2007; Stocker et al., 2014). The Himalayan region has been warming more rapidly over the past few decades compared to average global warming (IPCC, 2007; Shrestha, Gautam, & Bawa, 2012; Shrestha, Wake, Mayewski, & Dibb, 1999). Recent monthly mean and annual mean temperatures have broken previous records (GISTEMP Team, 2016; Hansen, Ruedy, Sato, & Lo, 2010), and Friedrich, Timmermann, Tigchelaar, Timm, and Ganopolski (2016) consider that current climate projections are possibly underestimated. Further evidence of this has been reported from all polar regions (Comiso & Hall, 2014; Pachauri et al., 2014), including the “third pole,” the Himalaya (Armstrong, 2010; Huettmann, 2012; Pachauri et al., 2014). Given the current governance of climate‐related issues, we have adopted a precautionary approach to our choice of climate change scenario and have chosen the representative concentration pathway 8.5 (RCP8.5, “business‐as‐usual”) as a future climate scenario, which we consider to be the most realistic for our study area.

We took an average of five downscaled general circulation models, namely ACCESS1‐0, BCC‐CSM1‐1, GISS‐E2‐R, MIROC‐ESM‐CHEM and MPI‐ESM‐LR, to reduce model‐wise variations (Beaumont, Hughes, & Pitman, 2008; Suwal & Vetaas, 2017). We predicted for a single worst case scenario (i.e., RCP8.5) and a single future period 2070 (average of 2060 to 2080; Hijmans et al., 2005).

### 2.6 Analysis, distribution model preparation, and validation

The values of all environmental variables were extracted at occurrence points, background data points, and lattice points (points that are arranged in a grid at 3 arc minutes distance in the study area, total = 177,938, on which current and future distributions were predicted). All the analyses were performed as a point‐based analysis (using environmental values extracted at points instead of raster files; e.g., Kandel et al., 2015; Regmi et al., 2018).

We used the following analytical path: We applied constrained principal component analysis (PCA) for the eastern and western regions’ climatic difference; Tukey's honest significant difference (HSD) test for climatic range differences for all 24 variables; multivariate analysis of variance (MANOVA) for niche differences between taxa; the “background test” to analyze niche similarity with available climate; and, finally, distributions of species were modeled with MaxEnt and Random Forest (details below). This was done with both climate datasets, that is, CHELSA‐ and WorldClim‐predictors.

The climatic similarity between the eastern region (of *M. assamensis* spp. a*ssamensis*) and western region (of *M. assamensis* spp. *pelops*) was evaluated using PCA in the *R* package *vegan* (Oksanen et al., 2013). An equal number of random points (15,000 for each region; note: Density of points is not equal here) was used from the eastern and western regions, on which raster values of nine least correlated topo‐climatic variables were extracted from raster files. Then, constrained PCA was performed on the values (separately for CHELSA and WorldClim‐predictors), and “region” was treated as a predictor to analyze climatic similarity between regions (999 permutation tests).

Post hoc Tukey's HSD with a 0.95 confidence interval was used (for occurrence data) to test the difference in the realized climate range of all variables (square root‐transformed) between the two subspecies. A variable range graph was plotted by standardizing all the variables to values between 0 and 2 to aid range comparisons between the taxa. A MANOVA (Pillai, 1985) was used to test whether the realized climatic niches of the two subspecies were statistically similar. We used a subset of nine selected independent variables (cf. above), and the taxa were coded as a fixed factor.

The background test evaluates whether the distribution (or niches) of two species is more or less similar than expected based on the environmental background of where they occur (Warren, Glor, & Turelli, 2010). This will indicate whether the realized niche of one subspecies is more or less similar to the realized niche of another subspecies based on the environmental conditions theoretically available to them (i.e., ignoring the barrier). In this test, we used the environment of the whole study area as background because *M. assamensis* ssp. *assamensis* is a descendant of *M. assamensis* ssp. *pelops*, which dispersed to new areas in the past and is not yet fully evolved into a new species. This asymmetric background test was performed for both taxa, and similarity measures *D* (Schoener, 1968) and *I* (Warren, Glor, & Turelli, 2008) are reported along with their respective statistics. This is an additional test to the MANOVA as MANOVA was used to test differences in the realized niche between taxa based on occurrence data, while the background test assesses whether the realized niche is more or less similar than random expectation given the climate of the study area.

To answer the third research question, species distribution models (SDMs) were developed using MaxEnt (Phillips, Anderson, & Schapire, 2006) and Random Forest (Breiman, 2001a; Liaw & Wiener, 2002) algorithms, which are among the most commonly used machine learning methods (Aguirre‐Gutiérrez et al., 2013; Mi, Huettmann, Guo, Han, & Wen, 2017). The models were fitted with binary response data (occurrence data with background data) in the *R* package *sdm* (Naimi & Araújo, 2016). Although it is claimed that both algorithms are not much affected by multicollinearity (Breiman, 2001a; Elith et al., 2011), the models were run on subsets of the nine least correlated predictor variables, because higher dimensionality may cause poor model extrapolation and transferability (Peterson, 2011; but see Breiman, 2001a,b)

Models were fitted separately for each subspecies with CHELSA‐ and WorldClim‐predictors. The models were set to the default settings, except replication, which was set as fivefold cross‐validation (CV) for 10 times, regularization multiplier, which was set to 1.0 for *M. assamensis* ssp. *assamensis* and 0.5 for *M. assamensis* ssp. *pelops* (based on AIC scores tested between 0 and 10 at 0.5 intervals in *ENMTools* (Warren & Seifert, 2011)) in MaxEnt, and using out‐of‐bag (OOB) sampling in Random Forest. Models were trained with 70% of binary response data and remained 30% was used for model evaluation. We used one threshold‐independent evaluation measure—area under the curve (AUC) of the receiver operating characteristic (ROC; Bradley, 1997; Hanley & McNeil, 1982) and two threshold‐dependent evaluation measures—true skill statistic (TSS; Allouche, Tsoar, & Kadmon, 2006) and omission error with “maximum sum of sensitivity and specificity” threshold.

Predictions from models were made on lattice files prepared as above from each run for each subspecies and separately for CHELSA‐ and WorldClim‐predictors. Future predictions from CHELSA‐predictor‐trained models were not performed because the future scenario of CHELSA is not available at the moment. The average of 50 replications (5 CV * 10 runs) predicting the relative index of occurrence (RIO) was used for further analysis. The average (of 50 replications) variable importance based on the AUC test score was extracted from MaxEnt and Random Forest models and illustrated graphically.

To plot a two‐dimensional realized climatic niche for each subspecies, we selected one temperature and one precipitation variable because they are the key dimensions of climatic niches (Bell et al., 2014; Margules et al., 1987; Vetaas, 2002). From the WorldClim‐predictors, the most important precipitation and temperature variables are bio18 and bio09, respectively, for both taxa. With the CHELSA‐predictors, bio18 was the most important precipitation variable in three of four cases (two taxa, two models), and hence, it was chosen. However, for the temperature variable, both bio03 and bio08 were top in two of four cases. For simplicity, we chose bio08 over bio03, because bio03 is more complex (ratio of bio02 and bio07) than bio08. The two selected variable sets were used to plot two‐dimensional realized climatic space with density isolines using the *R* package *ggplot2* (Wickham, 2010).

### 2.7 Analysis of prediction similarity of CHELSA and WorldClim‐predictors

The similarities in the predictions (Breiman, 2001b) from CHELSA‐ and WorldClim‐predictors were analyzed using both MaxEnt and Random Forest models from raster files (in ASCII format, prepared by inverse distance weighted method from RIO value) supplied to the *ENMTools* software (Warren et al., 2008). The range overlaps between taxa were analyzed with the respective threshold “maximum sum of sensitivity and specificity” (e.g., Jiménez‐Valverde & Lobo, 2007) from each model. The similarity of the predictions between eastern and western populations, and between MaxEnt and Random Forest for future geographical distributions, was estimated by *ENMTools* using two indices *D* and *I*. Both *D* and *I* indices range between 0 (no similarity) and 1 (identical prediction). The *D* and *I* are calculated by taking the difference between the relative indices of occurrence score for each grid cell (for details see Warren et al., 2008; Warren, Glor, & Turelli, 2009).

## 3 RESULTS

### 3.1 Climatic similarity between the eastern and western regions

The similarity analysis of the climatic conditions using constrained PCA shows significantly different climatic conditions between the eastern and western regions with both CHELSA‐ (*r* ^2^ = 0.194, *p *<* *0.001) and WorldClim‐predictors (*r* ^2^ = 0.198, *p *<* *0.001). The PCA plots show a partly overlapping distribution of points from the two regions (details in Supporting Information Figure S2). Although the overall climatic conditions are significantly different between the eastern and western regions, there are patches with similar climate among the two.

A “background test” was performed for each subspecies with respect to the total climatic background available to them (includes both eastern and western regions). The background test for the eastern population in ENMTools suggests that its realized climate is less similar to the background than random expectation (i.e., given the background climate available; CHELSA: *D* = 0.14, *p* < 0.05; *I* = 0.35, *p* < 0.05; WorldClim: *D* = 0.22, *p* < 0.05; *I* = 0.49, *p* < 0.05; Supporting Information Figure S3A), while the background test for the western population shows that the realized climate does not significantly differ from the background climate (CHELSA: *D* = 0.29, *p* > 0.05; *I* = 0.56, *p* > 0.05; WorldClim: *D* = 0.27, *p* > 0.05; *I* = 0.54, *p* > 0.05; Supporting Information Figure S3B).

### 3.2 Climatic niche overlaps between the two subspecies

**Figure 3:**
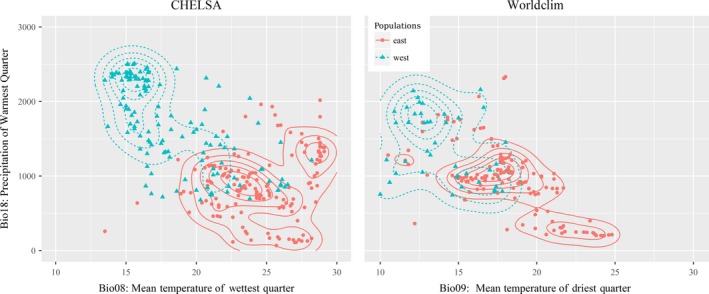
The two‐dimensional realized climatic niche (CHELSA: bio08 (mean temperature of wettest quarter) versus bio18 (precipitation of warmest quarter; left panel); WorldClim: bio09 (mean temperature of driest quarter) versus bio18 (right panel)) shows that the climatic niche of the western population (*Macaca assamensis* ssp. *pelops*) overlaps with the core climatic niche of the eastern population (*M. assamensis* ssp. *assamensis*) and the climatic niche of the eastern population overlaps a peripheral area of the climatic niche of the Western population

Tukey's HSD test reveals significantly different ranges of 21 variables between the two subspecies (except bio14, bio19, and aspect for CHELSA‐predictors, and bio12, bio16, and aspect for WorldClim‐predictors; Supporting Information Table S2 and Figure S4). The MANOVA test shows significantly different realized climatic niches between the two subspecies (CHELSA: Pillai's trace = 0.86882, *p *<* *0.001; WorldClim: Pillai's trace = 0.7629, *p *<* *0.001). The climatic niche difference is also visible in the two‐dimensional niche plots (Figure 3), where the density isolines of the two subspecies have a distinct orientation.

### 3.3 Potential distribution of sister taxa under current climatic conditions

**Figure 4:**
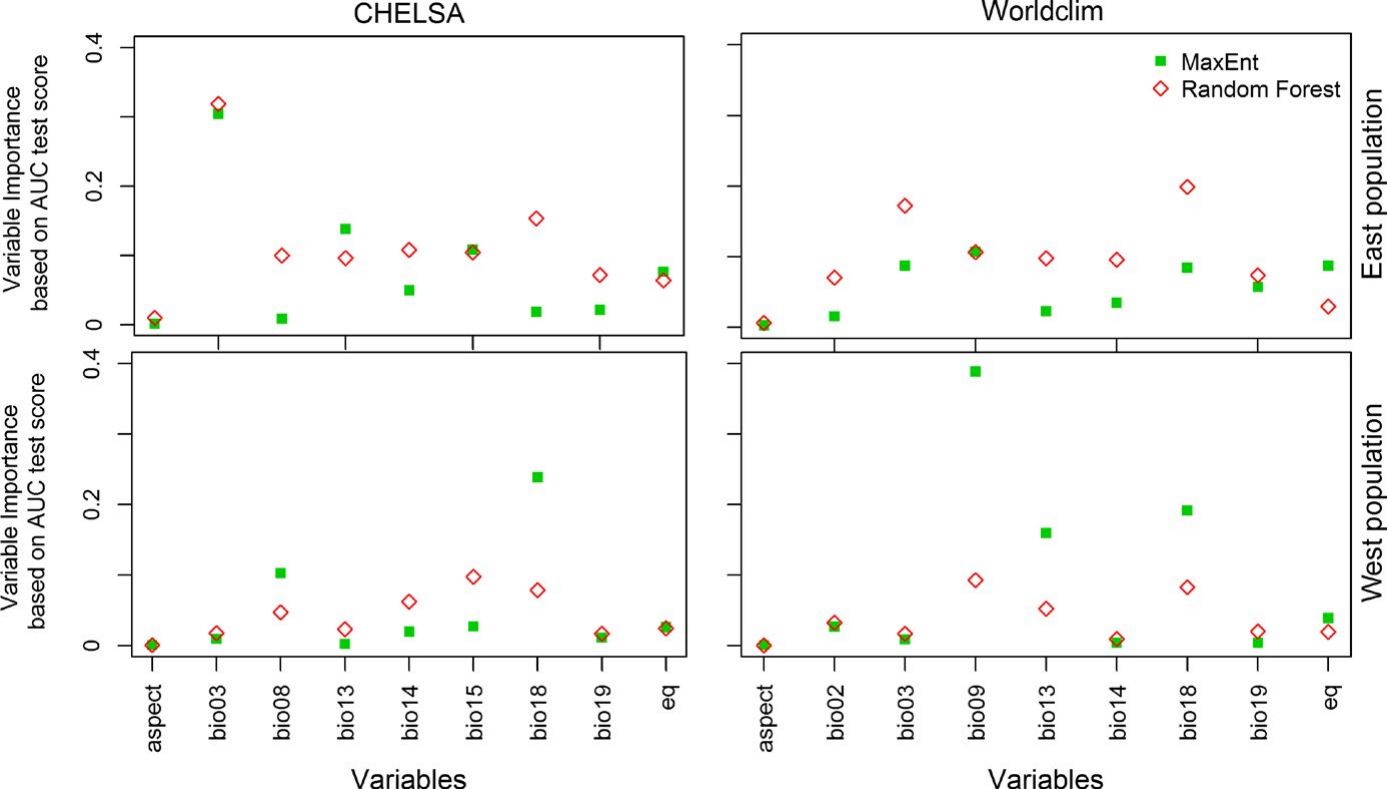
Variable importance based on area under the curve (AUC) test scores from MaxEnt and Random Forest models. Bio02 = mean diurnal range, bio03 = isothermality, bio08 = mean temperature of wettest quarter, bio09 = mean temperature of driest quarter, bio13 = precipitation of wettest month, bio14 = precipitation of driest month, bio15 = precipitation seasonality, bio18 = precipitation of warmest quarter, bio19 = precipitation of coldest quarter, eq = Ellenberg climatic quotient

The distribution models for the western population consistently have better AUCs, TSSs, and omission errors compared to the eastern population models (Table 1). The most important variable of the different analyses with respect to subspecies, methods, and climate data source varies with the analysis (details in Figure 4).

**Table 1:** Model performance measures of MaxEnt and Random Forest for two subspecies of *Macaca assamensis*

Method	AUC	TSS	Omission error (in %)	Subspecies
CHELSA				
MaxEnt	0.921	0.71	14.08	*M.a.assamensis*
Random Forest	0.930	0.73	12.78	*M.a.assamensis*
MaxEnt	0.992	0.94	2.41	*M.a.pelops*
Random Forest	0.989	0.92	4.48	*M.a.pelops*
WorldClim				
MaxEnt	0.924	0.74	12.84	*M.a.assamensis*
Random Forest	0.938	0.76	13.24	*M.a.assamensis*
MaxEnt	0.994	0.94	2.92	*M.a.pelops*
Random Forest	0.993	0.94	2.70	*M.a.pelops*

*Note*

Higher area under the curve (AUC) and true skill statistic (TSS) values indicate a better model, as do lower values of omission error.

**Figure 5:**
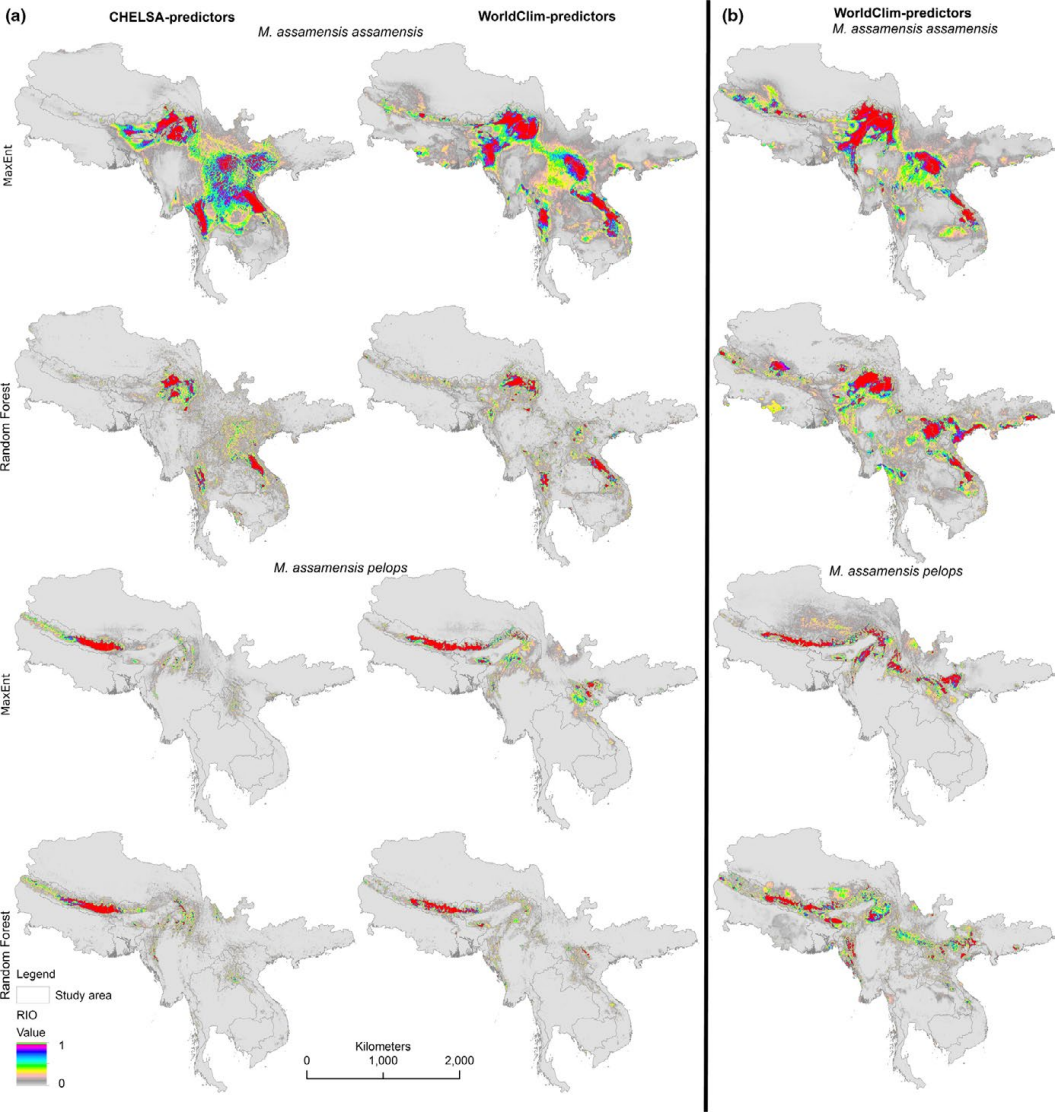
Potential distribution of two subspecies of *Macaca assamensis* based on MaxEnt and Random Forest models. The maps illustrate relative index of occurrence (RIO) predictions for *M. assamensis* ssp. *assamensis* and *M. assamensis* ssp. *pelops* distributions in (a) the current climate, using CHELSA‐predictors (left) and WorldClim‐predictors (right), and (b) a future (2070) climate scenario (from WorldClim‐predictors only)

**Figure 6:**
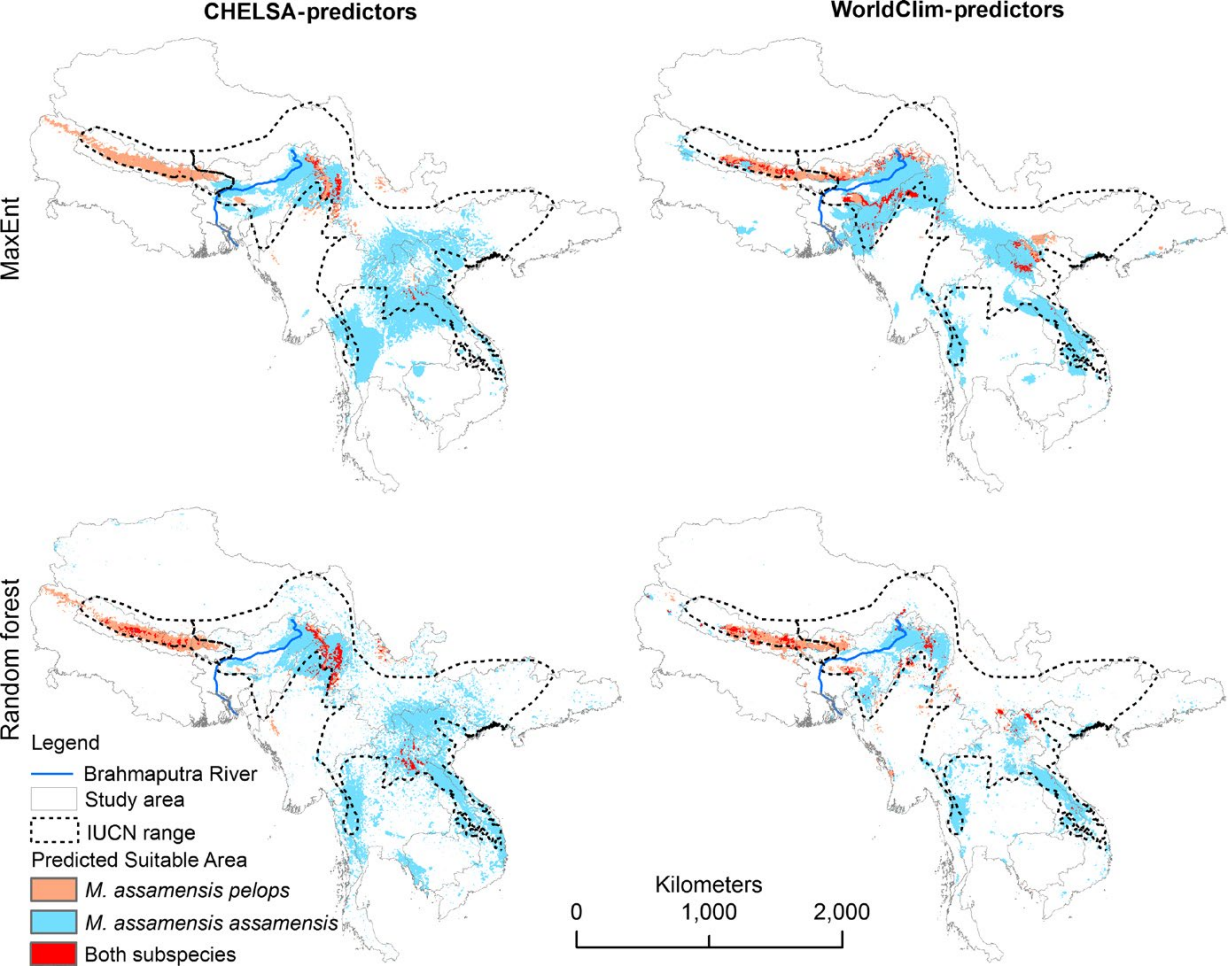
Potential area of distribution based on binary predictions (suitable/unsuitable) with maximum sensitivity plus specificity thresholds for CHELSA‐predictors and WorldClim‐predictors, as modelled by MaxEnt and Random Forest under current climate conditions. In the figure, the white background inside the study areas is predicted as not potential area. The IUCN range map was extracted from the IUCN Red List portal (http://www.iucnredlist.org), accessed on 27 November 2016. The figure implies uncertainty of predictions related to climate data source and modelling methods

The predicted potential distribution areas are wider than their currently known distribution areas for both subspecies (Figure 5a). This is particularly true for the eastern subspecies. Although the realized climatic niches of the two subspecies are statistically different, the distribution models show that the neighboring areas bordering the ranges of each and some core geography areas appear to be broadly suitable for both subspecies (Figures 5a and 6). The western area shows comparatively more potential area that could be occupied by the eastern population (Figure 5a). The result also agrees with the background test (cf. above). It suggests that both regions have some potential area for both subspecies.

The predicted overlaps of potential area between subspecies using an average of ‘maximum sum of sensitivity and specificity” threshold (Table 2) vary among methods and datasets. The overlap between subspecies is 1.6% and 4.8% for MaxEnt and Random Forest, respectively, for CHELSA‐predictors. Similarly, overlap of subspecies for WorldClim‐predictors is 5.6% and 6.9% for MaxEnt and Random Forest, respectively.

**Table 2:** The “maximum sum of sensitivity and specificity” threshold for MaxEnt and Random Forest for CHELSA and WorldClim data sources and the two subspecies of *Macaca assamensis* to estimate their range overlaps under the current climate

	CHELSA‐predictors		WorldClim‐predictors	
	MaxEnt	Random Forest	MaxEnt	Random Forest
*M. a*. ssp. *pelops*	0.055689	0.014217	0.049471	0.009709
*M. a*. ssp. *assamensis*	0.172274	0.005475	0.138802	0.007842
Average	0.113981	0.009846	0.094136	0.008775

MaxEnt models based on CHELSA‐predictors predict 6.6% more potential area for the eastern population and 3.6% more area for the western population compared to WorldClim‐predictors under the current climate. Random Forest models with CHELSA‐predictors predict 48.1% more potential area for the eastern population and 10.1% more area for the western population compared to WorldClim‐predictors.

### 3.4 The potential distribution of the two subspecies under a projected future climate

**Figure 7:**
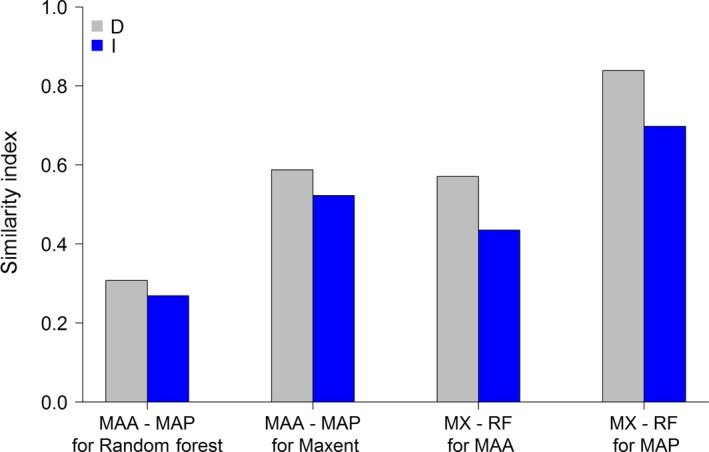
Graph of similarity indices D and I from ENMTools for predictions by MaxEnt and Random Forest for future climatic conditions with WorldClim‐predictors. The value 0 means completely dissimilar and 1 is totally identical conditions. The graph shows the predicted similarity between *Macaca assamensis* ssp. *assamensis* (MAA) and *M. assamensis* ssp. *pelops* (MAP) from Random Forest (RF) modelling (first pair of columns) and MaxEnt (MX; second pair of columns) and illustrates the prediction similarity between MaxEnt and Random Forest in reference to both taxa (third and fourth pairs of columns)

The comparison between MaxEnt and Random Forest on future potential areas for the two subspecies shows that predictions are method‐dependent. The similarity between future predictions (*D* and *I* similarity indices) by MaxEnt and Random Forest is between 43 and 84% (Figure 7), respectively. The similarity in the potential areas in the future climate for the eastern and western populations is between 26 and 58% (Figure 7). The predicted potential distribution using WorldClim‐predictors is depicted in Figure 5b. In the future projected climate, the number of potential patches is greater compared to current climatic conditions (Figure 5a,b). This suggests fragmentation of the potential area under future climate and may cause loss of connectivity between the patches, thus threatening the species survival and having implications for conservation.

### 3.5 Prediction similarity test of CHELSA‐ and WorldClim‐predictors

We created four models using the CHELSA‐predictors and another four using WorldClim‐predictors. The AUC is always marginally greater for the WorldClim‐predictors than for the CHELSA‐predictors. Similarly, based on the TSS scores, the WorldClim‐predictors are generally better than the CHELSA‐predictors (three of four models and one equal). The omission error supports two of four models for both data sources. Based on the majority of results from the AUC, TSS, and omission error, WorldClim‐predictors outperform the CHELSA‐predictors (Table 1).

## 4 DISCUSSION

### 4.1 Climatic similarity between eastern and western regions

PCA shows that climatic conditions in the eastern region and western region are different. The difference in climate is possibe because of the positions of landmasses. The eastern region has comparatively more area in the warmer south, whereas the western region has higher mountains, resulting in a colder climate. In the western region, about 75% of precipitation occurs during the monsoon period (June to September) while in the eastern region, about the same amount of precipitation falls between May and November (http://sdwebx.worldbank.org/climateportal).

Although the overall climate between the eastern and western regions is statistically different, both regions may have some patches that are climatically similar, for instance a river valley or mountain slope. Such areas are probably predicted as being suitable for both taxa in our models in both regions. This is supported by the background test. The available background environment is not significantly different from the environment of the western population. The western population, therefore, successfully colonized and established in the eastern region in the past when the zoogeographic barrier was not effective (Fooden, 1988).

### 4.2 Climatic niche overlaps between the two subspecies

The realized climatic ranges of most of the variables are significantly different between the eastern and western populations, as suggested by Tukey's HSD test (Supporting Information Table S2). The MANOVA also reveals that the climatic niches of the two taxa are significantly different, possibly due to local climatic context. It is obvious that when climates of two regions are significantly different, the realized climate niches of two taxa also show significant difference. It is the same case with two parapatric subspecies of *M. assamensis* in this study. This result aligns with previous findings, for instance the distinct realized niches of six different sister taxa of Hanuman Langur (*Semnopithecus* spp.) in Peninsular India (Chetan, Praveen, & Vasudeva, 2014), and the distinct distribution and different realized niches of a subspecies of Californian scrub jay (*Aphelocoma californica*) in Mexico (Peterson & Holt, 2003). These previous findings and our results suggest that closely related taxa do not necessarily have similar realized niches (but see Peterson, 2011). The macaque subspecies are geographically isolated and living in different climatic conditions, which may promote speciation (Matute, Novak, & Coyne, 2009; Schluter, 2009).

The combined two‐dimensional realized climatic niche of both subspecies (Figure 3) shows their distinct orientation. Many points (and isodensity lines too in the case of WorldClim‐predictors) of the western population overlap with the core area of the eastern population, while a few points of the eastern population overlap with the core realized climate niche of the western population. This suggests, assuming the occurrence data are representative, that although the climate of two regions is significantly different, the climate of the eastern population is comparatively more suitable for the western population than vice versa, as is also supported by the background test.

### 4.3 The potential distribution of both subspecies in the current climate

Based on the AUC values, all MaxEnt and Random Forest models are considered good (>0.9) and the TSS measures suggest that MaxEnt and Random Forest models for *M. assamensis* ssp. *pelops* are excellent (>0.9) and good (>0.7) for *M. assamensis* ssp. *assamensis* (Swets, 1988; Zhang et al., 2015). Our models can thus be considered valid and allow for good inference (Table 1). However, the omission error of both MaxEnt and Random Forest for the eastern population is notably high. This is probably because of the wide geographic distribution that is a challenge for the model‐training procedure (Franklin, Wejnert, Hathaway, Rochester, & Fisher, 2009; McPherson & Jetz, 2007; Suwal & Vetaas, 2017).

There are some model‐wise variations in the predicted potential distributions of both sister taxa. MaxEnt and Random Forest models suggest that there are more potential areas than are currently occupied or reported for both subspecies. In the absence of the true area occupied by the species, we could not accurately estimate the total potential area that is not occupied by them. The prediction maps (Figures 5 and 6) show that the eastern population has comparatively more potential area outside its currently known distribution, while the western population has fewer suitable areas beyond its currently reported localities. Some of the areas are beyond the IUCN range map of the species (Boonratana et al., 2008; Figure 6).

The IUCN range maps lack clear reproducible codes and are essentially based on expert knowledge of species occurrences and models. They do not use recent predictive modeling tools and documentation, and hence lack meaningful quantitative error estimates. Here, we produced, for the first time, a model‐based quantitative potential distribution map using the best‐available data for *M. assamensis*, which is more transparent and repeatable compared with expert maps. The IUCN range map of *M. assamensis* (Boonratana et al., 2008) is broader than the currently known distribution (Fooden, 1980, 1988; Timmins & Duckworth, 2013; Wada, 2005), particularly in the northern area. There are, however, a few occurrence points in Myanmar and Thailand that are outside the IUCN boundary and the range map is much wider than the climatically potential area predicted by our models. In contrast, Herkt, Skidmore, and Fahr (2017) demonstrated that their potential distribution map for bats in Africa was much larger than the IUCN‐expert map. We agree with their observation that the IUCN maps differ considerably from SDMs, but the IUCN maps are normally based on documented occurrences whereas SDMs often find the potential distribution based on predictor variables. SDMs can be complementary to the currently available IUCN species’ range maps; thus, they could aid species conservation by highlighting the potential range of a species (Herkt et al., 2017). If applied correctly, this approach can contribute to better species management and serve as an improved tool for future conservation in areas where human population pressures are rising steeply (Mace et al., 2010). This option is technically easy, but has been widely ignored for over a decade in the times of the Anthropocene.

*Macaca assamensis* is already categorized as “Near Threatened” by the IUCN, suggesting the need for much higher priority in its conservation. The IUCN has listed habitat destruction due to anthropogenic activities as the major threat to the species; other threats are alien invasive species in the habitat, hunting, and trapping (Gray et al., 2018). The predicted potential area—which is currently thought to be unoccupied—under current climate may allow the extension of their distribution or provide suitable sites for their translocation in the event that their current localities become subject to the above‐mentioned threats or any kind of disease or human–macaque conflict. Our findings and data have direct conservation implications such as prioritizing species‐specific conservation areas, formulating species management and conservation action plans, identifying potential translocation sites, and exploring potential areas for new populations. Our output is open access in the hope that other researchers and conservationists can test, re‐validate, and use our findings to the benefit of the macaques and better habitat conservation overall.

We acknowledge that land use and anthropogenic disturbances can shape the geographic distribution and realized niche size of species (e.g., Miller & McGill, 2017; Zhao et al., 2018). Landscapes fragmented by human land use can interrupt the connectivity between habitat patches, which has consequences for the dispersal of species (e.g., Miller & McGill, 2017). Additionally, species distribution and the realized niche of species are also defined by ecological processes including predator–prey relationships and availability of food (Cushman et al., 2010; Hutchinson, 1957). However, here we limited our scope of study to topo‐bioclimatic variables and employed widely used algorithms to initiate this discussion and assessment. This is because data about anthropogenic disturbance, food availability, biotic interactions, and other ecological processes are complicated to document, although land cover data are available for the current period. We overlaid a land cover map on the predicted potential distribution map for current climate (Supporting Information Figure S5). The maps show that some of the predicted potential areas lie outside the current forest area, and thus, those areas are unlikely to be inhabited by *M. assamensis* as it is primarily a forest species. We did not use the land cover data in our model preparation because we aimed to model the potential future distribution of the species, which requires predictable variables. At the moment, this is not easily achievable for land cover; however, climatic variables can predict reasonably. One of the potential consequences of not incorporating such variables in ENMs and SDMs is that the models may predict a larger realized niche and wider potential distribution than is reasonable (Zhao et al., 2018).

### 4.4 Potential distribution of the two subspecies under projected future climate

There is currently no good way to test whether a future prediction is accurate or not (Huettmann & Gottschalk, 2011). Typically, the validity of the prediction is estimated from performance measures of the models. Based on the AUC and TSS (Table 1), all of our models are “good,” allowing for robust inferences. However, there are some model‐wise discrepancies in their predictions (Figure 7). These problems are often tackled by making an ensemble of multiple models (Araújo & New, 2007; Regmi et al., 2018), but we did not do this here directly. Instead, we used one of the best algorithms in SDM (Aguirre‐Gutiérrez et al., 2013; Craig & Huettmann, 2009; Mi et al., 2017), and, due to bagging, Random Forest being an ensemble model (Breiman, 2001a).

Regardless of some geographic differences in the future predictions, a common trend seen in both models is that both subspecies will have more potential area in the future. We could not estimate the total area because we avoided using any thresholds from the relative index of occurrence (RIO) to convert the future prediction into suitable/unsuitable areas. Continuous RIO values incorporate the uncertainty directly to avoid both false‐positive as well as false‐negative errors (Guisan et al., 2013), but there is no way to verify the results. The predicted potential areas under future climate are, to some extent, outside the current geographical distribution of both subspecies. Accessibility of those areas and the migration capability of the species may be a topic of additional research; it is not ecologically sound to assume any type of migration, although it is commonly done. Here, disregarding any dispersal ability of the species, we only evaluated the potential distribution under a projected future climate, which can inform conservation policy for the species such as pro‐active planning for assisted migration or the allocation of potential areas to protected status.

### 4.5 Prediction similarity test on CHELSA‐ and WorldClim‐predictors

The comparative study of the modeling using climate data from two sources shows that results can depend on, and be sensitive to, the source of the climate data. From Tukey's HSD test, the list of variables whose ranges are statistically similar varies between the two sources of data. Likewise, the prediction maps show that the areas predicted depend on the climate data source. This result aligns with some previous findings (e.g., Bedia et al., 2013; Pliscoff, Luebert, Hilger, & Guisan, 2014). Based on the model performance measures (AUC, TSS, omission error) in this study, the CHELSA‐predictors are outperformed by WorldClim‐predictors by a marginal value (for nine of 12 variables, one is equal; Table 1). Our climate data findings for Asia do not agree with previous findings by Bedia et al. (2013) and Bobrowski and Schickhoff (2017), who conclude that the WorldClim dataset is inferior to others and that it leads to misleading distribution models by consistently overpredicting the potential distribution (Bedia et al., 2013; Bobrowski & Schickhoff, 2017).

## 5 CONCLUSIONS

The climatic niches of two subspecies of *Macaca assamensis* are not as similar as expected by phylogenetic niche conservatism. Given the taxonomic subspecies would be valid; the difference in climatic niches between the subspecies is most probably due to the different climate of the eastern and western regions. Species distribution models predict unique as well as some common potential distribution areas for both subspecies. The potential geographic localities are predicted to change with contemporary anthropogenic climate change, which has implications for their conservation management.

## AUTHOR CONTRIBUTIONS

The study was planned and carried out by MKS. Occurrence data were contributed by GRR, FH provided help with the text, Random Forest modeling, predictions, and biogeography interpretations. The data handling, modelling, GIS works and tests were carried out by MKS. ORV supervised this work. We acknowledge provided software support by MaxEnt, Salford Systems Ltd, R and others.

## DATA ACCESSIBILITY

We used open‐access data from WorldClim (http://worldclim.org/version1; https://doi.org/10.1002/joc.1276) and CHELSA (http://chelsa-climate.org/; https://doi.org/10.1038/sdata.2017.122). The variables we prepared (ABT, EQ, slope, aspect), occurrence data, and our model outputs have been made open access via a university repository http://hdl.handle.net/1956/16960.
