# Supplementary material for: Parapatric subspecies of Macaca assamensis show a marginal overlap in their predicted potential distribution: Some elaborations for modern conservation management
Source: Ecol Evol. 2018 Sep 4;8(19):9712–27. doi: 10.1002/ece3.4405 (PMC6202702; doi:10.1002/ece3.4405)
Supplement: Supplementary file 1 [file ECE3-8-9712-s001.docx]

Supplementary Table S1: Variable details and selected list of CHELSA and Worldclim-predictors through variable selection

| SN | Variable | Details | CHELSA VIF selected | Worldclim VIF selected |
| --- | --- | --- | --- | --- |
| 1 | bio01 | Annual Mean Temperature |  |  |
| 2 | bio02 | Mean Diurnal Range (Mean of monthly (max temp - min temp)) |  | √ |
| 3 | bio03 | Isothermality (BIO2/BIO7) (* 100) | √ | √ |
| 4 | bio04 | Temperature Seasonality (standard deviation *100) |  |  |
| 5 | bio05 | Maximum Temperature of Warmest Month |  |  |
| 6 | bio06 | Mininmum Temperature of Coldest Month |  |  |
| 7 | bio07 | Temperature Annual Range (BIO5-BIO6) |  |  |
| 8 | bio08 | Mean Temperature of Wettest Quarter | √ |  |
| 9 | bio09 | Mean Temperature of Driest Quarter |  | √ |
| 10 | bio10 | Mean Temperature of Warmest Quarter |  |  |
| 11 | bio11 | Mean Temperature of Coldest Quarter |  |  |
| 12 | bio12 | Annual Precipitation |  |  |
| 13 | bio13 | Precipitation of Wettest Month | √ | √ |
| 14 | bio14 | Precipitation of Driest Month | √ | √ |
| 15 | bio15 | Precipitation Seasonality (Coefficient of Variation) | √ |  |
| 16 | bio16 | Precipitation of Wettest Quarter |  |  |
| 17 | bio17 | Precipitation of Driest Quarter |  |  |
| 18 | bio18 | Precipitation of Warmest Quarter | √ | √ |
| 19 | bio19 | Precipitation of Coldest Quarter | √ | √ |
| 20 | abt | Annual BioTemperature |  |  |
| 21 | eq | Ellenberg Climatic Quotient | √ | √ |
| 22 | Elev | SRTM Digital Elevation Model |  |  |
| 23 | Slope | Topographic Slope |  |  |
| 24 | Aspect | Topographic Aspect | √ | √ |

Supplementary Figure S1: Variable clusters to select the least correlated variables. The subplot (A) is from CHELSA-predictors and (B) from-WorldClim predictors.

*
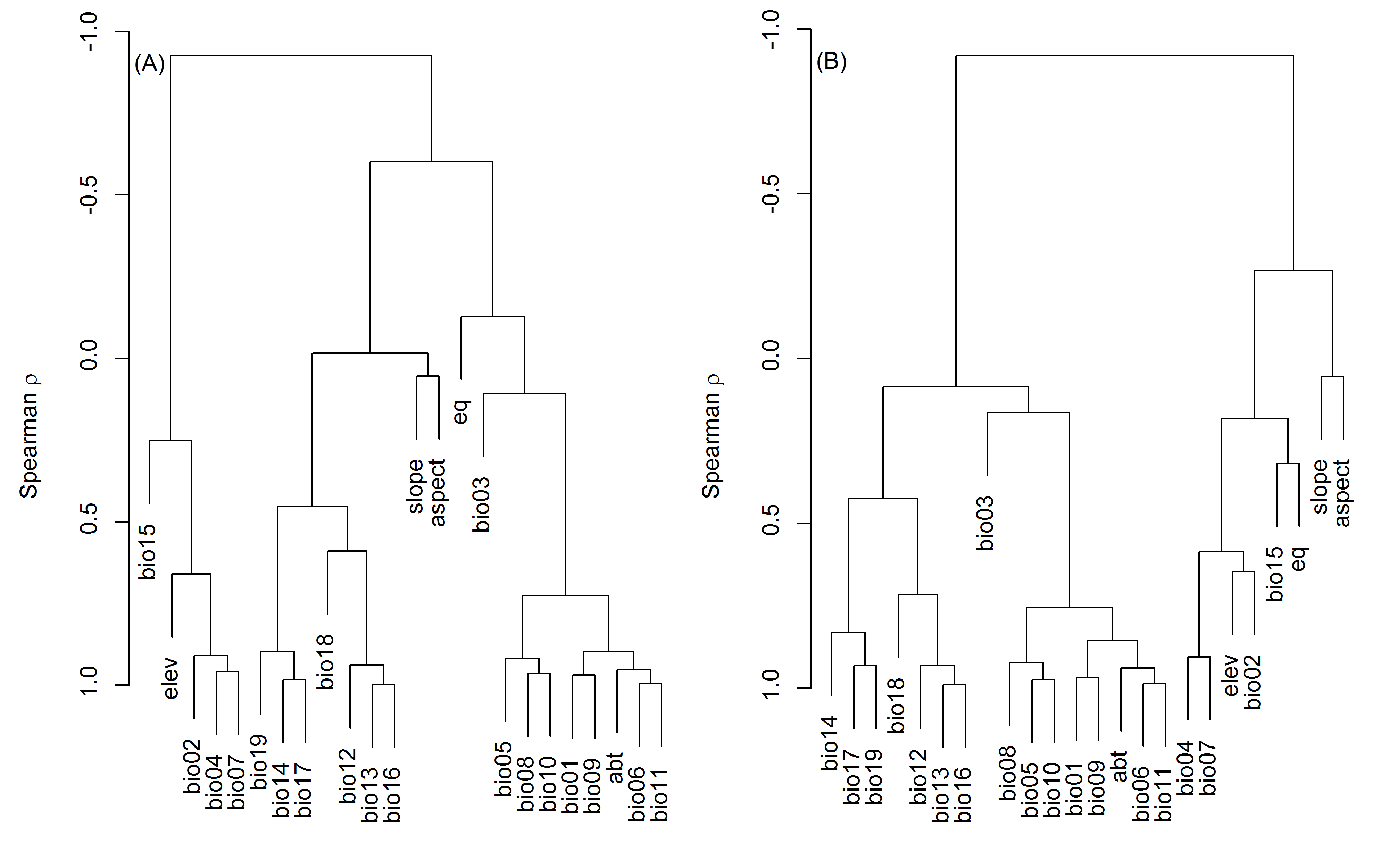
*

Supplementary Figure S2: PCA plots show partial overlap of points from eastern and western region of respective eastern population and western population. The climatic condition between them are significantly different.


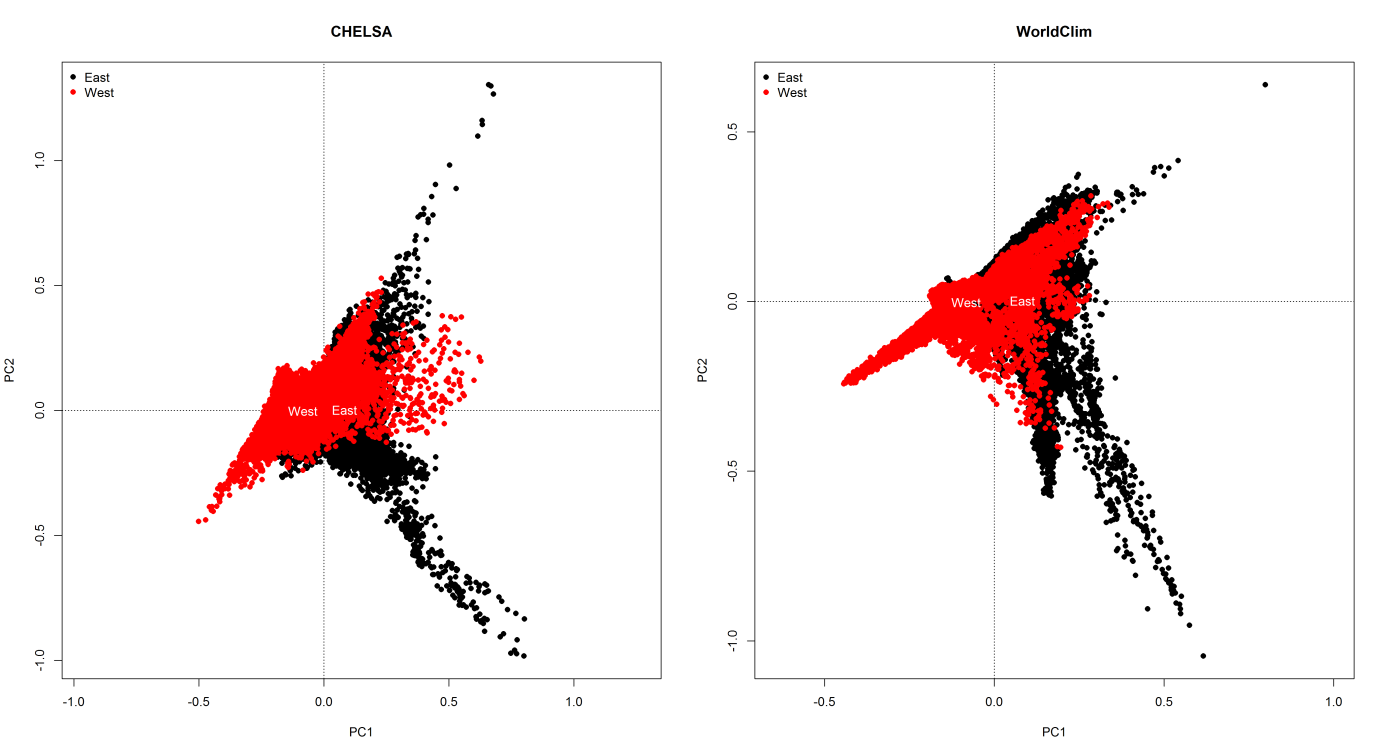


Supplementary Figure S3: Background test for both taxa, with CHELSA- predictors (A) and WorldClim-predictors (B)

| (A) | (B) |
| --- | --- |
| 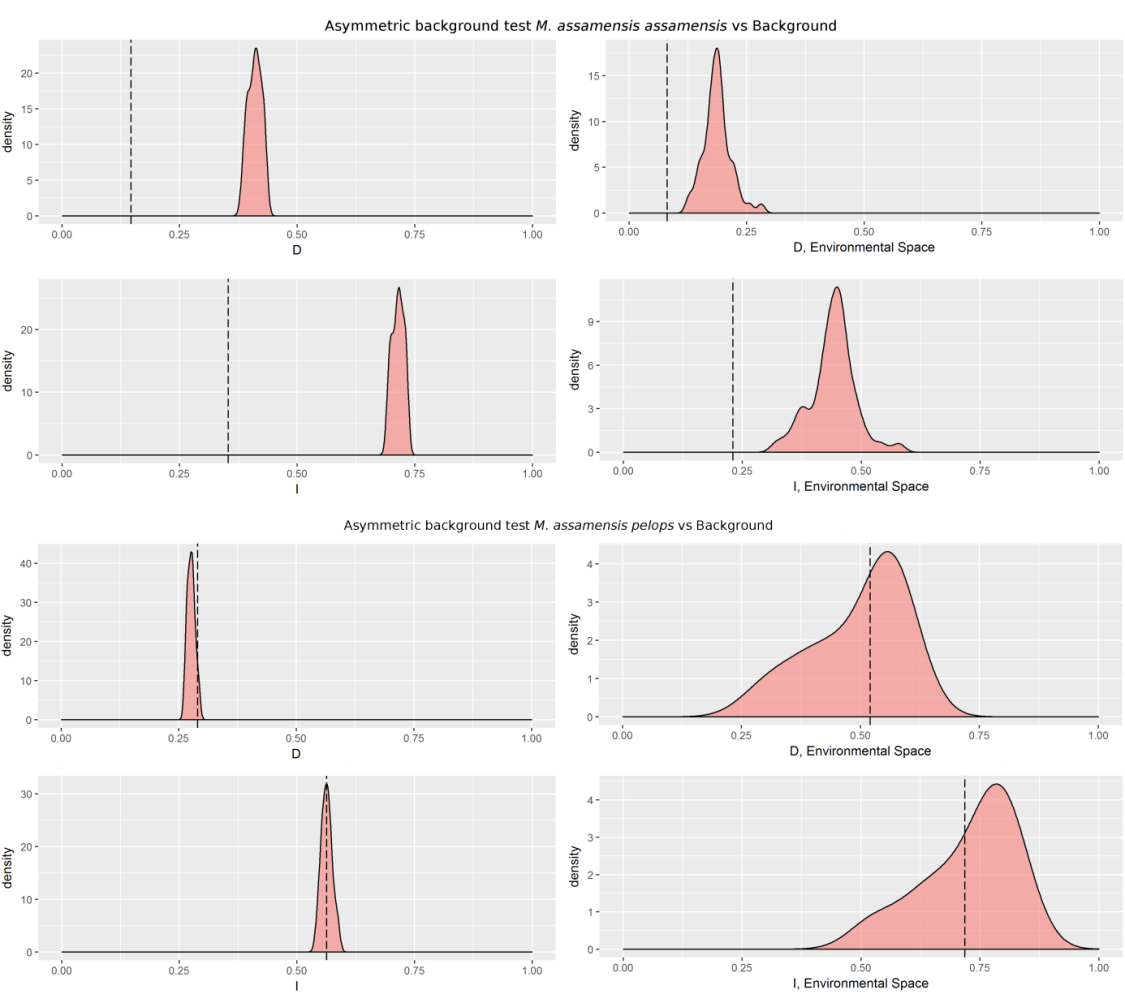 | 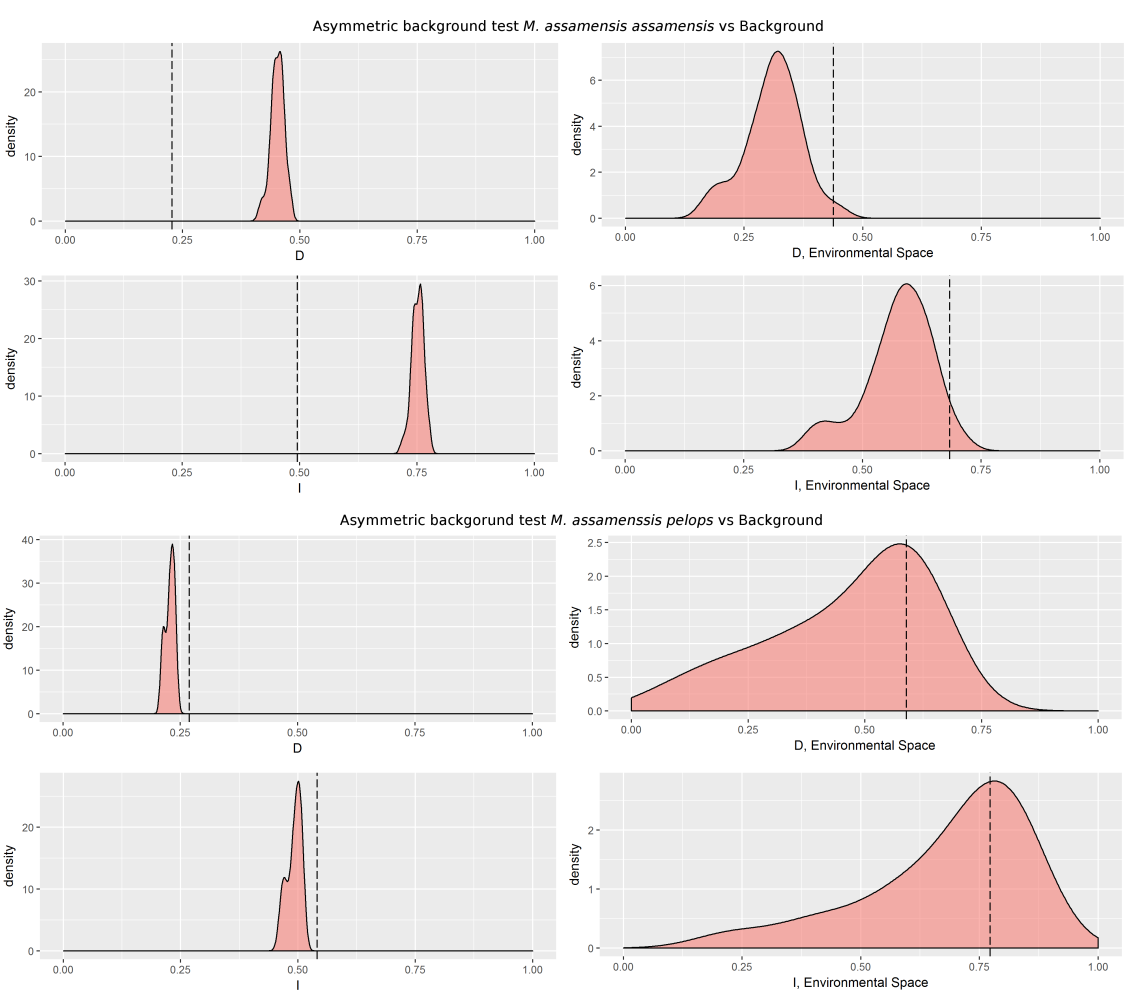 |

Supplementary Table S2: Tukey's Honesty Significant Test results. Negative values first adjusted to positive, then Square root transformed, afterward they were readjusted

| **CHELSA Predictors** | | | | | | |
| --- | --- | --- | --- | --- | --- | --- |
| S.N. | Variables | Difference in means | Lower | Upper | *p*-adjusted | Significant Difference at 0.05 |
| 1 | bio01 | -1.275 | -1.369 | -1.182 | 0 | TRUE |
| 2 | bio02 | -0.032 | -0.056 | -0.007 | 0.012 | TRUE |
| 3 | bio03 | -0.389 | -0.416 | -0.362 | 0 | TRUE |
| 4 | bio04 | 4.338 | 3.93 | 4.747 | 0 | TRUE |
| 5 | bio05 | -0.953 | -1.016 | -0.891 | 0 | TRUE |
| 6 | bio06 | -1.281 | -1.545 | -1.017 | 0 | TRUE |
| 7 | bio07 | 0.228 | 0.19 | 0.265 | 0 | TRUE |
| 8 | bio08 | -0.753 | -0.823 | -0.684 | 0 | TRUE |
| 9 | bio09 | -1.56 | -1.673 | -1.447 | 0 | TRUE |
| 10 | bio10 | -0.807 | -0.875 | -0.739 | 0 | TRUE |
| 11 | bio11 | -1.968 | -2.1 | -1.835 | 0 | TRUE |
| 12 | bio12 | 6.202 | 4.62 | 7.784 | 0 | TRUE |
| 13 | bio13 | 5.589 | 4.787 | 6.39 | 0 | TRUE |
| 14 | bio14 | -0.165 | -0.371 | 0.04 | 0.114 | FALSE |
| 15 | bio15 | 0.988 | 0.921 | 1.054 | 0 | TRUE |
| 16 | bio16 | 8.107 | 6.822 | 9.391 | 0 | TRUE |
| 17 | bio17 | -0.433 | -0.794 | -0.072 | 0.019 | TRUE |
| 18 | bio18 | 13.971 | 12.396 | 15.545 | 0 | TRUE |
| 19 | bio19 | 0.306 | -0.117 | 0.729 | 0.155 | FALSE |
| 20 | abt | -1.27 | -1.363 | -1.178 | 0 | TRUE |
| 21 | eq | -0.9 | -1.043 | -0.758 | 0 | TRUE |
| 22 | elev | 20.395 | 18.52 | 22.269 | 0 | TRUE |
| 23 | slope | 1.722 | 1.464 | 1.98 | 0 | TRUE |
| 24 | aspect | 0.689 | -0.166 | 1.544 | 0.114 | FALSE |
|  |  |  |  |  |  |  |
| **Worldclim Predictors** | | | | | | |
|  | Variables | Difference in means | Lower | Upper | *p*- adjusted | Significant Difference at 0.05 |
| 1 | bio01 | -3.38824 | -3.66782 | -3.10866 | 0 | TRUE |
| 2 | bio02 | -0.68121 | -0.81894 | -0.54349 | 0 | TRUE |
| 3 | bio03 | -0.36612 | -0.42366 | -0.30859 | 0 | TRUE |
| 4 | bio04 | 8.85985 | 7.609009 | 10.11069 | 0 | TRUE |
| 5 | bio05 | -2.90961 | -3.15378 | -2.66544 | 0 | TRUE |
| 6 | bio06 | -3.40276 | -3.71532 | -3.0902 | 0 | TRUE |
| 7 | bio07 | -0.27808 | -0.42718 | -0.12899 | 0 | TRUE |
| 8 | bio08 | -2.43361 | -2.6654 | -2.20181 | 0 | TRUE |
| 9 | bio09 | -3.24695 | -3.49678 | -2.99712 | 0 | TRUE |
| 10 | bio10 | -2.66778 | -2.90216 | -2.4334 | 0 | TRUE |
| 11 | bio11 | -3.44897 | -3.71239 | -3.18555 | 0 | TRUE |
| 12 | bio12 | -1.13118 | -2.27218 | 0.009813 | 0.052 | FALSE |
| 13 | bio13 | 1.149147 | 0.49093 | 1.807365 | 0.001 | TRUE |
| 14 | bio14 | -0.61549 | -0.80016 | -0.43082 | 0 | TRUE |
| 15 | bio15 | 0.616267 | 0.52841 | 0.704124 | 0 | TRUE |
| 16 | bio16 | 0.91449 | -0.11516 | 1.944142 | 0.082 | FALSE |
| 17 | bio17 | -0.67811 | -0.97938 | -0.37684 | 0 | TRUE |
| 18 | bio18 | 5.648939 | 4.366375 | 6.931502 | 0 | TRUE |
| 19 | bio19 | -1.15078 | -1.44499 | -0.85658 | 0 | TRUE |
| 20 | abt | -1.05519 | -1.14185 | -0.96854 | 0 | TRUE |
| 21 | eq | -0.58193 | -0.68915 | -0.47471 | 0 | TRUE |
| 22 | elev | 20.39472 | 18.5204 | 22.26905 | 0 | TRUE |
| 23 | slope | 1.722303 | 1.464181 | 1.980426 | 0 | TRUE |
| 24 | aspect | 0.757372 | -0.10596 | 1.620705 | 0.085 | FALSE |

Supplementary Figure S4: Variables ranges overlap between two subspecies depicted in modified boxplots. The horizontal line inside boxes represent the median, box ranges from 5 to 95th percentile and Whisker lines of boxplot represent minimum and maximum of data. In the figure subplot ‘A’ is from CHELSA-predictors and ‘B’ from WorldClim-predictors.


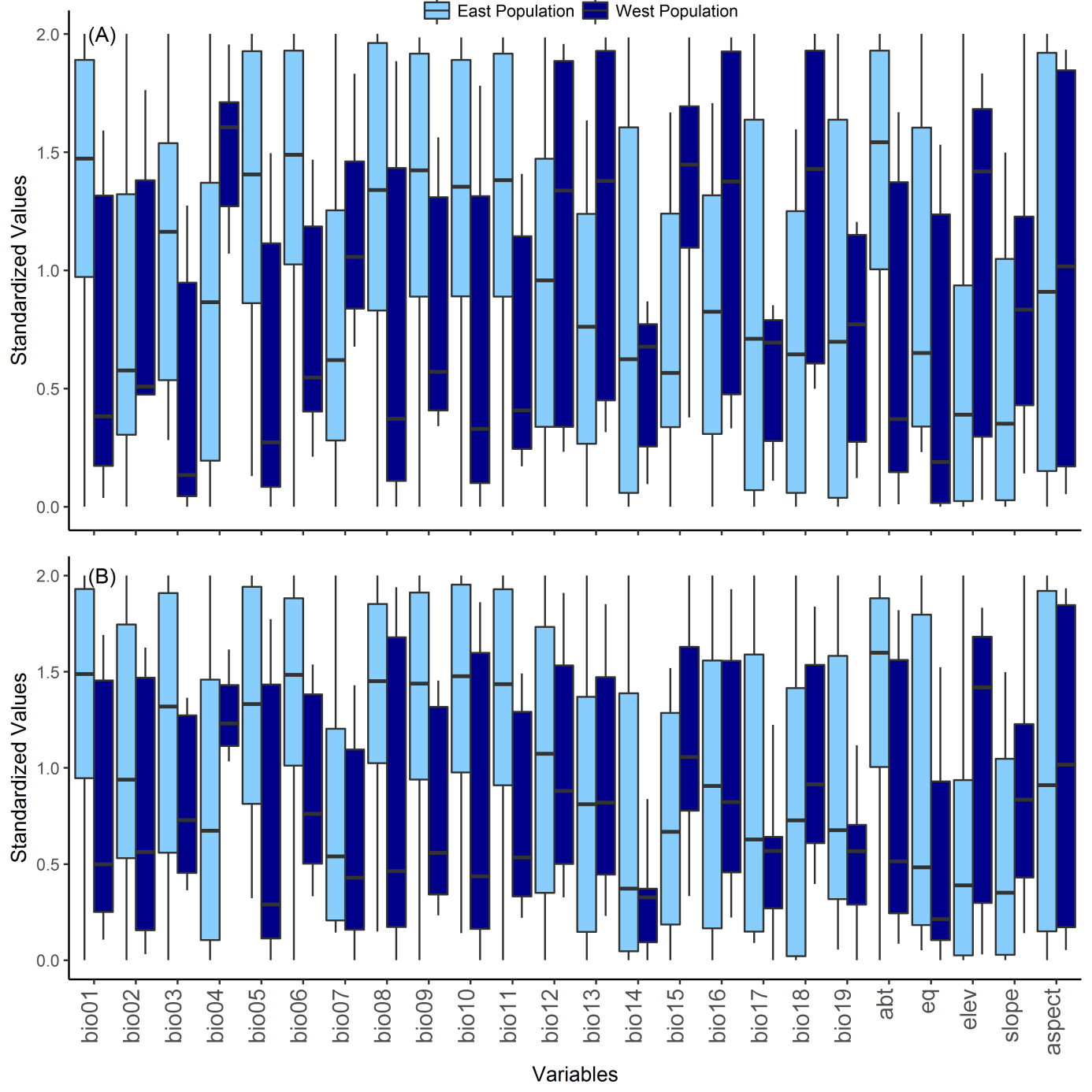


Supplementary Figure S5: Land cover map overlay on model prediction for current climate. The land cover map is obtained from Broxton *et. al.* (2014). Here, the “forest” on map refers to deciduous broadleaf forest, deciduous needle leaf forest, evergreen broadleaf forest, evergreen needle leaf forest, mixed forests and woody savannas. We used single legend for all forest to ease illustration. In the map, the darker green area is where both predicted potential distribution and forest overlaps, white area inside the study area is non-forest area and predicted unsuitable area.


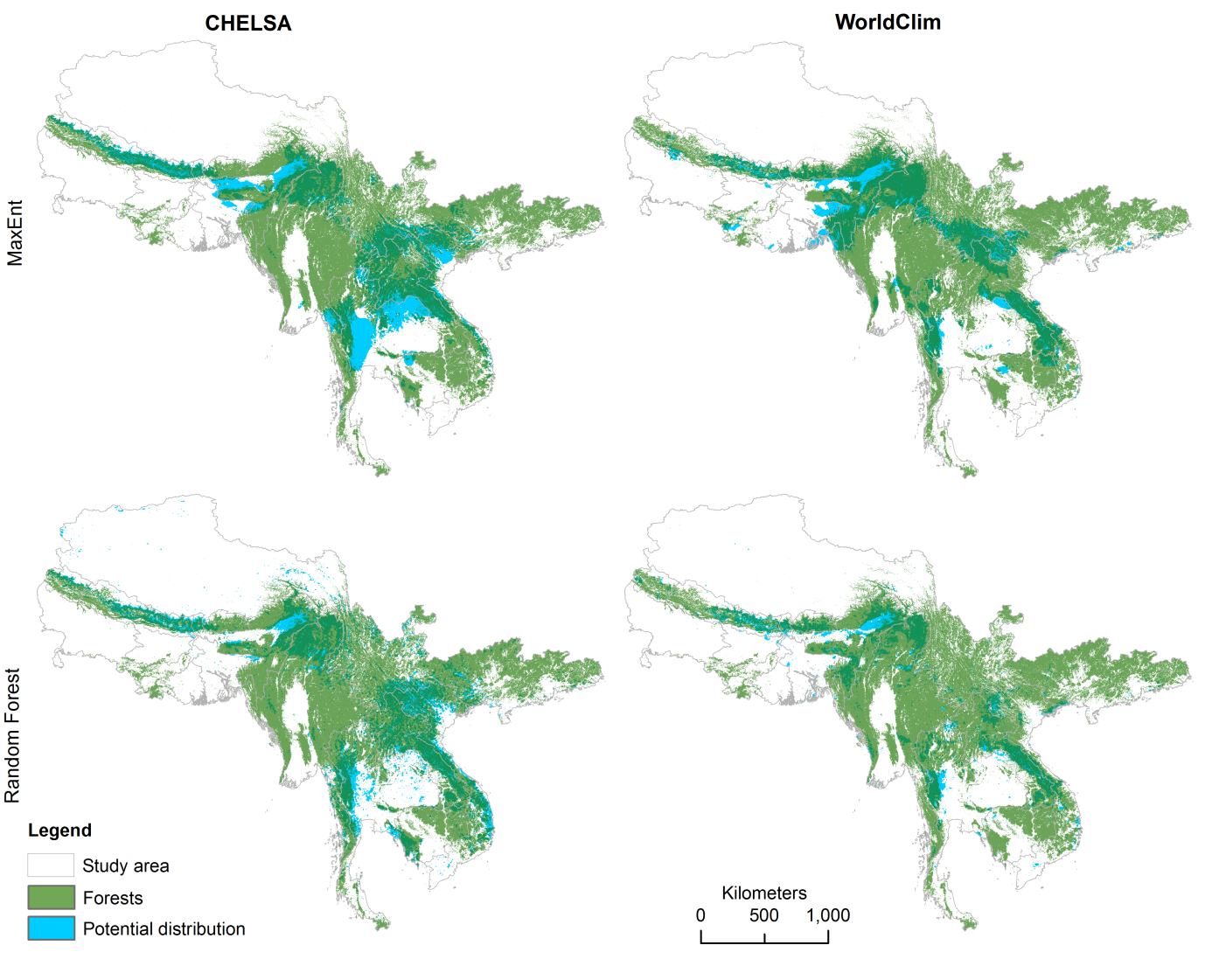


Reference:

Broxton, P.D., Zeng, X., Sulla-Menashe, D., Troch, P.A., (2014) A Global Land Cover Climatology Using MODIS Data. J. Appl. Meteor. Climatol., 53, 1593 ─ 1605. doi: <http://dx.doi.org/10.1175/JAMC-D-13-0270.1>, URL https://landcover.usgs.gov/global_climatology.php
